# AQP1 Is Up-Regulated by Hypoxia and Leads to Increased Cell Water Permeability, Motility, and Migration in Neuroblastoma

**DOI:** 10.3389/fcell.2021.605272

**Published:** 2021-02-11

**Authors:** Zihe Huo, Mihai Lomora, Urs Kym, Cornelia Palivan, Stefan G. Holland-Cunz, Stephanie J. Gros

**Affiliations:** ^1^Department of Pediatric Surgery, University Children’s Hospital Basel, Basel, Switzerland; ^2^Department of Clinical Research, University of Basel, Basel, Switzerland; ^3^Department of Physical Chemistry, University of Basel, Basel, Switzerland

**Keywords:** tumor cell motility, tumor cell migration, membrane water transport, hypoxic microenvironment, aquaporin 1, HIF—hypoxia inducible factor, neuroblastoma

## Abstract

The water channel aquaporin 1 (AQP1) has been implicated in tumor progression and metastasis. It is hypothesized that AQP1 expression can facilitate the transmembrane water transport leading to changes in cell structure that promote migration. Its impact in neuroblastoma has not been addressed so far. The objectives of this study have been to determine whether AQP1 expression in neuroblastoma is dependent on hypoxia, to demonstrate whether AQP1 is functionally relevant for migration, and to further define AQP1-dependent properties of the migrating cells. This was determined by investigating the reaction of neuroblastoma cell lines, particularly SH-SY5Y, Kelly, SH-EP Tet-21/N and SK-N-BE(2)-M17 to hypoxia, quantitating the AQP1-related water permeability by stopped-flow spectroscopy, and studying the migration-related properties of the cells in a modified transwell assay. We find that AQP1 expression in neuroblastoma cells is up-regulated by hypoxic conditions, and that increased AQP1 expression enabled the cells to form a phenotype which is associated with migratory properties and increased cell agility. This suggests that the hypoxic tumor microenvironment is the trigger for some tumor cells to transition to a migratory phenotype. We demonstrate that migrating tumor cell express elevated AQP1 levels and a hypoxic biochemical phenotype. Our experiments strongly suggest that elevated AQP1 might be a key driver in transitioning stable tumor cells to migrating tumor cells in a hypoxic microenvironment.

## Introduction

AQP1 was the first molecular water channel to be described (Preston et al., 1992). It is located in the plasma membrane and physiologically predominantly found in red blood cells, kidney, lung, vascular endothelium, brain and eye (King et al., 2004). Recent discoveries on involvement of AQP1 in cell proliferation and migration have suggested that AQP1 could play a key role in tumor biology (Papadopoulos et al., 2008; Verkman et al., 2008). AQP1 has been shown to be expressed in proliferating malignant tumor cells as well as to play an important role in micro-vessel formation (Endo et al., 1999; Moon et al., 2003; Saadoun et al., 2005). A possible functional importance of AQP1 in tumor biology has been suggested regarding tumor-associated edema, facilitated tumor angiogenesis and endothelial cell migration, tumor cell extravasation and metastasis (Saadoun et al., 2005; Hara-Chikuma and Verkman, 2006; Verkman et al., 2008). AQP1 has been reported to be associated with a poor prognosis, especially in later stages of colon cancer, lung cancer and breast cancer (Otterbach et al., 2010; Machida et al., 2011; Yoshida et al., 2013). In these stages, hypoxia within the solid tumor is increasing. Cancer cells are characterized by dysregulated cell proliferation, and the blood vessels that form within solid tumors are often structurally and functionally abnormal, resulting in severe hypoxia (Semenza, 2012). Adaptation of cancer cells to the hypoxic microenvironment is regulated through physiological responses to hypoxia that are mediated by hypoxia-inducible factors (HIF), namely HIF-1α and HIF-2α. Due to these responses, hypoxic cancer cells can acquire enhanced invasive and metastatic properties as well as resistance to chemotherapy and radiation therapy, which together constitute the lethal cancer phenotype (Semenza, 2012). A hypoxia-dependent up-regulation of AQP1 has been demonstrated for endothelial cells in the rodent lung (Echevarria et al., 2007). In mouse endothelial cells and Schwann cells, in a model mimicking facial nerve injury, it has been suggested that the up-regulation of AQP1 occurs in a HIF-1α-dependent manner (Abreu-Rodriguez et al., 2011; Zhang et al., 2013). There is also evidence that HIF-1α facilitates up-regulation of other aquaporins, namely AQP4 and AQP9, in a rat model of traumatic brain injury (Ding et al., 2009). This could suggest that AQP1 is regulated by mechanisms within the hypoxic tumor microenvironment.

The presence of AQP1 and its impact on tumor progression and clinical impact in neuroblastoma has not been addressed. Data from the R2: microarray analysis and visualization platform suggest a correlation between AQP1 expression and reduced overall survival^1^. It is hypothesized here that by alteration of AQP1 expression cells can facilitate the transmembrane water transport leading to cell expansion and cell shape changes that further migration (Papadopoulos et al., 2008). It has been shown in an AQP1 knock-out breast cancer model that AQP1 gene deletion reduces breast tumor growth and lung metastasis (Esteva-Font et al., 2014). AQP1 has been associated with early organ development during embryogenesis specifically in kidney, lung and salivary gland (Devuyst et al., 1996; Horster, 2000; de Paula et al., 2020). In migrating Chinese hamster ovary (CHO) cells AQP1 polarizes to the lamellipodia and AQP1 deletion severely impairs migration (Saadoun et al., 2005). A further model on how water channels can contribute to cell migration was proposed by Stroka et al. (2014) and is called the osmotic engine model. Accordingly, an increase of water in- and out-flux during cell motion can enhance cell velocity. The objectives of this study were to determine whether AQP1 expression in neuroblastoma is dependent on hypoxia, to demonstrate whether AQP1 is functionally relevant for migration, and to further define AQP1-dependent properties of the migrating cells.

## Materials and Methods

### Choice of Cell Lines

**FIGURE 1:**
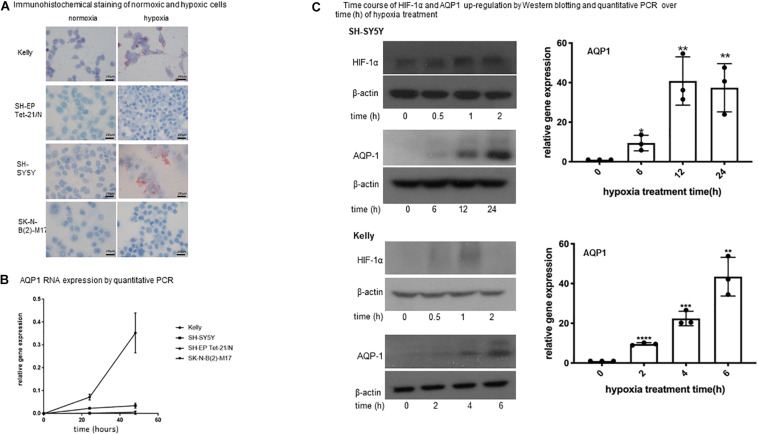
Expression of AQP1 in neuroblastoma cells under hypoxia**. (A)** Immunohistochemical staining of normoxic and hypoxic cells. Exposure of neuroblastoma cell lines Kelly, SH-SY5Y, SK-N-B(2)-M17 and SH-EP Tet-21/N to hypoxia (1% O_2_) for 48 h leads to an increased APQ1 protein expression (red staining) in Kelly and to a lesser extent in SH-SY5Y cells. SK-N-B(2)-M17 and SH-EP Tet-21/N cells do not show any expression (microscopy at x100). The experiment was performed in quadruplicate and performed twice. **(B)** AQP1 mRNA expression by quantitative PCR. Relative expression of AQP1 mRNA increases with time under hypoxia in Kelly and to a lesser extent in SH-SY5Y cells. SK-N-B(2)-M17 and SH-EP Tet-21/N cells only show an insignificant increase (values at 0 h compared with 48 h: Kelly *p* < 0.005, SH-SY5Y *p* < 0.05, SK-N-B(2)-M17 ns, SH-EP Tet-21/N ns). The experiment was performed in triplicate and repeated. One-way Anova was performed with GraphPad Prism7 after confirming normal distribution followed by Dunnet’s post-test. The error bars represent one standard deviation. **(C)** Time course of HIF-1α and AQP1 up-regulation by Western blotting and quantitative PCR over time (h) of hypoxia treatment. **(C)** Shows the time course (in hours) of AQP1 and HIF-1α protein and AQP1 mRNA increase upon exposure to hypoxia treatment in SH-SY5Y and Kelly cells. While HIF-1α protein increases quickly upon hypoxic stimulation in both cell lines, the reaction time of AQP1 protein occurrence varies between cell lines. AQP1 expression in Kelly cells has started already after 2 h and increases until 6 h, while SH-SY5Y start producing AQP1 later, after around 6 h of treatment, and peak 24 h after onset of hypoxia. AQP1 protein production is consistent with AQP1 mRNA expression. In both cell lines production of AQP1 is following expression of HIF-1α. The experiment was repeated twice. One-way Anova was performed with GraphPad Prism7 after confirming normal distribution followed by Dunnet’s post-test. The error bars represent one standard deviation.

For the first experimental part we have chosen to show data of the four cell lines Kelly, SH-SY5Y, SK-N-B(2)-M17 and SH-EP Tet-21/N in order to include basic and diverse cell lines. SK-N-BE(2)-M17 is a cell line that was derived by sub cloning from the SK-N-BE(2) neuroblastoma cell line which itself was established from the bone marrow after repeated courses of chemo- and radiotherapy (Biedler and Spengler, 1976). SH-EP Tet-21/N was derived from the non-NMYC amplified SH-EP cell line (Lutz et al., 1996, 1998). Experiments on SK-B-E(2)-M17 and SH-EP Tet-21/N were included to investigate a diversity of neuroblastoma cell lines. The functionally relevant experiments were performed with Kelly and SH-SY5Y cells, which we believe to be the most representative for neuroblastoma. Kelly and SH-SY5Y cell lines are widely used throughout the literature and present standard cell lines used for neuroblastoma experiments, culture models, for differentiation experiments and for animal models. Kelly, SH-SY5Y and SK-N-B(2)-M17 have a neuroblastic (N) phenotype while SH-EP features a substrate adherent (S) phenotype (Baumann Kubetzko et al., 2004; Walton et al., 2004; Ross and Spengler, 2007; Muhlethaler-Mottet et al., 2011). They express AQP1 to different degrees in normoxia (Figure 1) and are heterogeneous regarding NMYC expression. NMYC expression of SH-EP Tet-21/N cells can be regulated by adding tetracycline to the culture medium. In the presence of tetracycline NMYC is not expressed (NMYC off) while NMYC is expressed in cells cultured in medium without tetracycline. Kelly cells show NMYC expression while SH-SY5Y cells do not in regular medium (Supplementary Data). For the functional experiments and inhibition experiments SH-SY5Y cells were favored because they express little AQP1 in normoxia and more in hypoxia. Thus, AQP1 expression can be modified in either direction and cells do not express NMYC under regular culturing conditions.

Nevertheless, the high intra- and inter-tumor heterogeneity of neuroblastoma tumors and diversity of clinical presentation of the disease, changes of tumor attributes induced by previous therapies e.g., at time of relapse, present great challenges when evaluating experiments with neuroblastoma cell lines and need to be treated with awareness.

### Cell Culture

Neuroblastoma Kelly, SH-SY5Y, SK-N-B(2)-M17 (all European Collection of Authenticated Cell Cultures (ECACC)/Sigma-Aldrich, Munich, Germany) and SH-EP Tet-21/N cells (reported by Lutz et al., 1996, 1998, kindly provided by G. Eschenburg, Hamburg) were cultivated in Roswell Park Memorial Institute (RPMI) media containing 10% fetal calf serum (FCS). If possible, aliquots of early passages (Moon et al., 2003; Saadoun et al., 2005; Verkman et al., 2008) after purchase were used for all experiments. All cells were cultured in a humidified atmosphere at 37°C either in air with 5% CO_2_ under normoxic or with 5% CO_2_/1–5% O_2_ balanced with N_2_ under hypoxic conditions. For inhibition of AQP1 tetraethylammonium (TEA) was added to the medium to a final concentration of 100 μM (Brooks et al., 2000). For inhibition of HIF-1α and HIF-2α HIF-1α inhibitor KC7F2 (Sigma-Aldrich/Merck, Germany) and HIF-2α inhibitor HIF-2 Antagonist 2 (Sigma-Aldrich/Merck, Germany) were used at final concentrations of 50 and 20 μM respectively after dose titration (data not shown).

### Immunostaining

Cells were seeded in chamber slides (BD-Bioscience, Falcon, United States) and grown for 48 h at 37°C with 5% CO_2_ (normoxic conditions) or with 5% CO_2_/1% O_2_ balanced with N_2_ (hypoxic conditions). The cells were then fixated with 4% paraformaldehyde and washed thrice with PBS-Tween solution. Immunhistochemistry staining was performed using the HRP-AEC-System from R&D Systems (Minneapolis, United States) with polyclonal rabbit anti-AQP1 antibody (Merck Millipore, Germany) at a dilution of 1:400 and counterstained with Mayer’s hematoxylin solution (Spitalpharmazie, Basel). As control, sections were incubated with antibody diluent (DAKO, Denmark) without primary antibody at 4°C overnight and then treated as other samples. For immunofluorescence staining slides were fixated with 4% paraformaldehyde and permeabilized with 0.2% Triton X-100 in phosphate buffered solution. Blocking was performed with 3% bovine serum albumin in phosphate buffered solution with Tween-20 for 1 h at room temperature. Primary antibody against AQP1 was used as described above. Detection was achieved with goat anti-rabbit IgG (H + L) cross-adsorbed secondary antibody Alexa Fluor 555 or 647 (Invitrogen, Thermo Fisher Scientific Inc., Waltham, United States) respectively. ProLong^®^ Gold Antifade Mountant with DAPI (Life Technologies, Thermo Fisher Scientific Inc., Waltham, United States) was used for nuclear staining and mounting. Control sections were incubated with secondary antibody control. Imaging was performed on an Olympus BX43 microscope using CellSens software and Zeiss LSM 710 Rocky using Zen software. Cytoskeleton immunofluorescence staining was performed with Alexa Fluor^TM^ 647 Phalloidin (Invitrogen, Massachusetts, United States).

### RNA Isolation/cDNA Synthesis/qPCR

Cells were harvested, washed with ice-cold PBS and then lysed in the buffer RLT Plus (QIAGEN). RNA isolation was subsequently performed using the RNeasy Plus Mini (QIAGEN, Cat.No. 74134) or Micro (QIAGEN, Cat.No. 74034) Kit, for more or less then 5 × 10^5^ cells, respectively. RNA isolation was performed according to the manual, and RNA was eluted in 30 or 14 μl nuclease-free water, respectively. RNA concentration was determined using a Colibri Microvolume Spectrometer (BioConcept AG). CDNA synthesis was performed using the GoScript^TM^ Reverse Transcription System (Promega, Cat.No. A5000). A Biometra T-Personal Thermal Cycler (Analytik Jena, Germany) was used.

Quantitative PCR was performed using the FastStart Universal SYBR Green Master (Rox) (Roche, Cat.No. 4913850001). AQP1, HIF-1α, HIF-2α, phosphoglycerate kinase 1 (PGK1) and 18S specific primers (Microsynth, Switzerland) were used for amplification of cDNA. Primer pairs were designed according to exon junction span using the clone manager software (Sci-Ed Software). Per reaction, 5 μl Master Mix were mixed with primers fwd and rev (0.5 μM), 1 μl of cDNA template or water in NTC, and water to a final volume of 10 μl. 18S was used a reference gene. reactions were performed in triplicates in MicroAmp^TM^ Optical 384-Well Reaction Plates (Applied Biosystems, Cat.No. 4309849) in a ViiA 7 Real-Time PCR System (Applied Biosystems) using the associated software. Data were analyzed using the 2^−ΔΔCt^ method for graphs in Figure 1C and the 2^−ΔCT^ method × 1,000 in all other graphs and depicted in Excel (Microsoft Office 2010) and GraphPad Prism 7. Relative expression was calculated against expression of 18s. AQP1 expression levels have previously been evaluated by RNAseq in a panel of commonly used cell lines that include SH-SY5Y cells. These data are available at https://www.proteinatlas.org/ENSG00000240583-AQP1/cell#rna.

### Western Blot Analysis

Cells were grown to confluence under either normoxic or hypoxic conditions. Confluence was reached between 48 and 72 h. We did not observe a difference between normoxic and hypoxic conditions in reaching confluence. They were lysed with radioimmunoprecipitation assay buffer (Sigma-Aldrich, St. Louis, MO, United States). The protein detection was performed using the BCA Protein Assay Kit (Thermo Fisher Scientific Inc., Waltham, MA, United States). Twenty microgram of protein was dissolved on a 10% SDS-polyacrylamide gel electrophoresis and blotted to nitrocellulose membranes. After blocking with 5% dried milk for 1 h at room temperature, immunoblotting against AQP1 was done using polyclonal rabbit anti-AQP1 antibody (Merck Millipore, Germany) diluted at 1:1,500 and HIF-1α antibody (BD Biosciences, New Jersey, United States) diluted at 1:500. Anti-β-actin antibody (Cell Signaling Technology, Massachusetts, United States) diluted at 1:2,000 served as a loading control. Super Signal West Dura Extended Duration Substrate (Thermo Fisher Scientific Inc., Waltham, MA, United States) was used for detection.

### Transfection

Neuroblastoma SH-SY5Y cells were transfected using Nucleofector^TM^ Technology with Cell Line Nucleofector^TM^ Kit V (Lonza, Basel, Switzerland) with piLenti-siRNA-GFP (abmGood, Richmond, CA) for AQP1. Transfected cells were selected with puromycin treatment and intensity of GFP expression. AQP1 expression knockdown was confirmed by western blotting. Human AQP1 cDNA was cloned into the lentivirus vector pCDH-CMV-MCS-EF1-copGFP (System Biosciences, CA, United States). A 293T cell-based viral expression system using AQP1 and control plasmids and △R8.74, VSV-G and Rev with Xfect transfection reagent (TakaraBio, Shiga, Japan) was used according to the manufacturers’ guidelines. SH-SY5Y cells were infected with the lentivirus containing AQP1 cDNA. The GFP (green fluorescent protein)-positive cells were sorted by flow cytometry. The over-expression of AQP1 was confirmed in by qPCR and western blotting. Clones showing the highest down-regulation of AQP1 as well as the highest over-expression of AQP1 were chosen for further experiments.

### Stopped-Flow Spectroscopy

Cells were cultured under normoxic or hypoxic conditions for 48 h, harvested and adjusted to 3.5 × 10^6^ cells/mL in phosphate buffered saline (PBS). The function of AQP1 expression in SH-SY5Y cells was also evaluated as a response to a rapid change in osmotic pressure, by following the 90° scattered light, at a wavelength of 600 nm, using a stopped-flow apparatus (Bio-Logic SAS, France). Specifically, detached SH-SY5Y cells (AQP1 low or AQP1 high) were repeatedly mixed in a stopped-flow mixing chamber with 300 mM mannitol and the light scattering signal was monitored over time at room temperature. Experimental curves were represented as the average of three series, each of minimum of ten injections. Data were fitted according to a single exponential function, and the corresponding rate constants including standard error of estimate (SEE) were determined using the Biokine 32 V5.80 software (Bio-Logic Science Instruments, France).

### *In vitro* Wound Healing

Cells were cultured as confluent monolayers, synchronized in 1% fetal bovine serum for 24 h, and wounded by removing a defined 300–500 μm strip of cells across the well with a standard 200 μl pipette tip (Lee et al., 2000). The wounded monolayers were washed twice to remove non-adherent cells. Wound healing was quantified as the average linear speed of the wound edges over 24 h. In addition to using serum deprivation by synchronizing cells in 1% fetal bovine serum, wild type and AQP1-modified cells were compared regarding proliferation and no differences were found.

### Modified Migration Assay

Tumor cell migration through a microporous membrane was assessed using a Boyden transwell system (8 μm pore size, Corning Costar, NY, United States). Before seeding, transwell-systems were incubated in RPMI-media containing 2% FCS for 30 min at room temperature. Two hundred microliter RPMI-media with 2% FCS containing dissociated cells (3 × 10^4^ per well) was added to the upper insert of the chamber. In the bottom chamber, 500 μl RPMI-media with 10% FCS was added. Twenty-four hours after seeding, cells of the upper and lower chambers were harvested and placed on ice. Messenger RNA was retrieved and further processed as described above. Experiments were performed in triplicates and repeated twice. The experiment was also carried out seeding an equal number of both SH-SY5Y-AQP1 KD and regular SH-SY5Y cells. For depiction purposes, the entire membrane was preserved after the above described experiment, embedded in TissueTek OCT (Sakura, Japan), and snap frozen.

### Statistical Analysis

Data were analyzed using the SPSS version 23.0 (SPSS, Chicago, United States), Excel and GraphPad Prism 7 Software. For significance testing the two-sided *t*-test or analysis of variance with a *post hoc* test was used. *P*-values less than 0.05 were defined as significant. The error bars in all bar plots represent one standard deviation.

## Results

### AQP1 Expression Is Up-Regulated by Hypoxia

AQP1 expression of neuroblastoma cell was evaluated by immunohistochemistry, quantitative PCR and western blotting under normoxic and hypoxic conditions. Exposure of neuroblastoma cell lines Kelly, SH-SY5Y, SK-N-B(2)-M17, and SH-EP Tet-21/N to hypoxia led to an increase of AQP1 expression detected by immunohistochemistry in Kelly and to a lesser degree in SH-SY5Y cells (Figure 1A). Only Kelly cells showed a visible expression of AQP1 under normoxia. The same could be seen regarding AQP1 mRNA in quantitative PCR analysis over 48 h (Figure 1B). While AQP1 mRNA production under hypoxia in Kelly cells matched protein expression, SH-SY5Y cells showed AQP1 mRNA increase to a lesser extent. In SK-N-B(2)-M17 and SH-EP Tet-21/N cells exposure to hypoxia did not lead to major changes in AQP1 mRNA or protein expression and AQP1 could still not be detected in cells by immunohistochemistry or western blotting after 24 or 48 h. Kelly and SH-SY5Y cells were then closely monitored regarding the time-course of protein up-regulation of AQP1 and its relation to HIF-1α up-regulation (Figure 1C). We show that HIF-1α protein is already increasingly expressed after 30 min peaking after 2 h. Six hours after this HIF-1α increase we observe an increase of AQP1 protein expression in SH-SY5Y cells, in Kelly cells after 2–4 h. This indicates that AQP1 expression could be regulated by HIF-1α, as has been previously proposed (Abreu-Rodriguez et al., 2011).

### Functional Properties of AQP1 Expressing Cells

**FIGURE 2:**
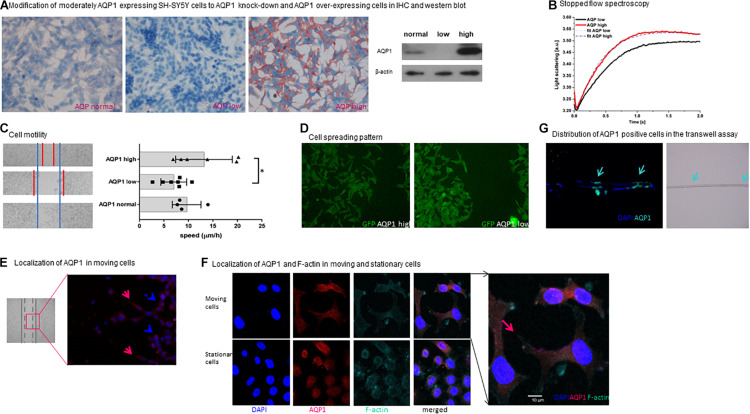
Functional properties of AQP1 expressing cells**. (A)** Modification of moderately AQP1 expressing SH-SY5Y cells to AQP1 knock-down and AQP1 over-expressing cells in IHC and western blot. Modification of SH-SY5Y cells results in an AQP1 knock-down (AQP1 low) and over-expressing cell line (AQP1 high). This was confirmed by immunohistochemistry (red staining of AQP1) and by western blot. Staining was performed in duplicate and repeated, blotting was performed twice. **(B)** Stopped flow spectroscopy. The kinetics of the initial increase of the curves confirm a faster reaction of AQP1 high cells against AQP1 low cells to the osmotic stimulus due to increased water channel function. The consecutive decrease of the curves is due to volume regulation mechanisms of the cells, again faster in the AQP1 high cells compared to the AQP1 low cells. This results in a higher rate constant for AQP1 high cells than AQP1 low cells: k_APQ__1__high_ = 3.27 ± 0.033 s^–1^ and k_APQ__1__low_ = 2.02 ± 0.007 s^–1^, respectively. Experimental curves are represented as the average of three series, each with a minimum of ten injections. Details regarding the analysis are given in the section “Materials and Methods”. **(C)** Cell motility. The left panel shows representative images of the migration progress of cells 24 h after the defined scratch in the wound healing assay using SH-SY5Y neuroblastoma cells. The blue lines represent the set border of the regular cell line (lower image), the red markings the margins of the AQP1 low (middle image) and AQP1 high cell (upper image) lines in bright field microscopy. The right panel depicts the speed of closure of the gap in μm/h, confirming the visual impression that closure of the gap by AQP1 high cells is increased and reduced in the AQP1 knock-down. Experiments were performed in quadruplicate and repeated twice. One-way Anova was performed followed by Tukey multiple comparison test using GraphPad Prism7. Normal distribution was confirmed. The error bars represent one standard deviation. **(D)** Cell spreading pattern. AQP1 high neuroblastoma cells (upper panel) show a distinct morphological pattern when closing the wound. They are more spread out and form more lamellipodia compared to AQP1 low neuroblastoma cells (lower panel). GFP expression of cells is unspecific and due to transfection. Experiment was performed in quadruplicate and repeated twice. **(E)** Localization of AQP1 in moving cells. Immunofluorescence staining of AQP1 (red) and cell nuclei (DAPI blue) shows that in the area in which cells migrate toward the gap in the wound healing assay, AQP1 is located in the forward moving part of the cell (marked by red arrows) and in the lamellipodia. Cells, that do not move (marked by blue arrow), have a round shape and AQP1 is localized around the nuclei. Staining was performed in duplicate and repeated twice. **(F)** Localization of AQP1 and F-actin in moving cells. In confocal fluorescence microscopy after AQP1 and F-actin staining AQP1 and actin are located in the lamellipodia (marked by red arrow), giving evidence of the cytoskeletal changes of the migrating SH-SY5Y neuroblastoma cells. Staining was performed in quadruplicate and performed twice. **(G)** Distribution of AQP1 positive cells in the transwell assay. Examination of the 8 μm pore membrane (right panel, pores are marked by arrows) of a transwell migration assay with a FCS gradient using SH-SY5Y cells captures AQP1 (green) expressing cells in immunofluorescence staining gathered around the pores (marked with arrows) on both sides of the membrane in the progress of migrating through the pores. AQP1 negative cells (blue, only positive for DAPI nuclei staining) are localized in other areas of the membrane. This was performed once to depict cell transit through membrane.

In order to compare functional properties of neuroblastoma cells that express different levels of AQP1, knock-down and over-expressing SH-SY5Y cells were constructed. Protein expression levels were confirmed using immunohistochemistry and western blot (Figure 2A). Stopped-flow spectroscopy was established to analyze the functionality of the introduced AQP1 water channel proteins. The reaction to the osmotic stimulus of cells expressing fewer AQP1 water channels was slower than that of cells with a high expression of AQP1 water channels (Figure 2B, rapid initial phases). Once exposed to the osmotic stimulus, a faster increase in the light scattering is observed in AQP1 over-expressing SH-SY5Y cells (AQP1 high) compared to AQP1 knock-down SH-SY5Y cells (AQP1 low). This results in a higher rate constant for AQP1 high cells than AQP1 low cells: k_APQ__1__high_ = 3.27 ± 0.033 s^–1^ and k_APQ__1__low_ = 2.02 ± 0.007 s^–1^, respectively (Figure 2B). A faster increase in the light scattering is associated with faster cell shrinkage due to a higher water efflux.

SH-SY5Y cells were filmed in confocal microscopy under the osmotic stimulus demonstrating the enormous cell shape changes associated with their movement through confined spaces (Supplementary Video 1). It is well-known that such shape changes require substantial water flux across the cell membrane (Saadoun et al., 2005). The stopped flow analysis (Figure 2B) demonstrates that the membrane AQP1 water channels that were introduced by transfection into SH-SY5Y cells are functional, and thus allow substantial water fluxes and likely facilitate the shape changes required for movement through narrow spaces.

When examining cell motility in the migration assay, it was observed that modified AQP1-high cells close the gap much faster than AQP1 knockdown cells (Figure 2C). This shows that AQP1 indeed furthers the ability of the tumor cells to migrate. The observed difference between AQP1-normal and AQP1-high cells is not statistically significant. These differences in the ability to migrate are strengthened by observation of the cells’ migration pattern. After stimulation of the cells to move and close the defined gap, we observed two distinctly different migration patterns. In an initial descriptive observation (Figure 2D) we find that cells with a low AQP1 expression gather more closely together and do not easily detach from each other, whereas cells with a high AQP1 expression detach more easily and form more lamellipodia. This demonstrates a higher migratory potential of cells with high AQP1. AQP1 is a membrane protein and in moving cells its expression is observed especially in the lamellipodia which are the forward moving parts of the cells (Figure 2E). Moving cells form lamellipodia while stationary cells do not. Figure 2F depicts such a migrating cell with co-staining of cytoskeleton protein (F-actin) and AQP1, revealing that AQP1 expression is especially located in the lamellipodia, while the cytoskeleton is reshaped by F-actin (Figure 2F). This strengthens the hypothesis that AQP1 polarization to the forward moving part of the migrating cell first leads to an increased water influx and thus enables actin restructuring in the front part of the cell and shrinkage in the back part of the cell (Papadopoulos et al., 2008). This mechanism enhances the forward movement and cell migration.

Performing a transwell migration assay with a FCS gradient we observed that mostly AQP1 positive cells localized around the pores of the transwell membrane (Figure 2G). This suggests that the migrating cells, specifically those cells in the vicinity to the pores, are the ones with a higher APQ1 protein expression, which facilitates transgression of the pores.

### Attributes of Tumor Cells That Actually Migrate

**FIGURE 3:**
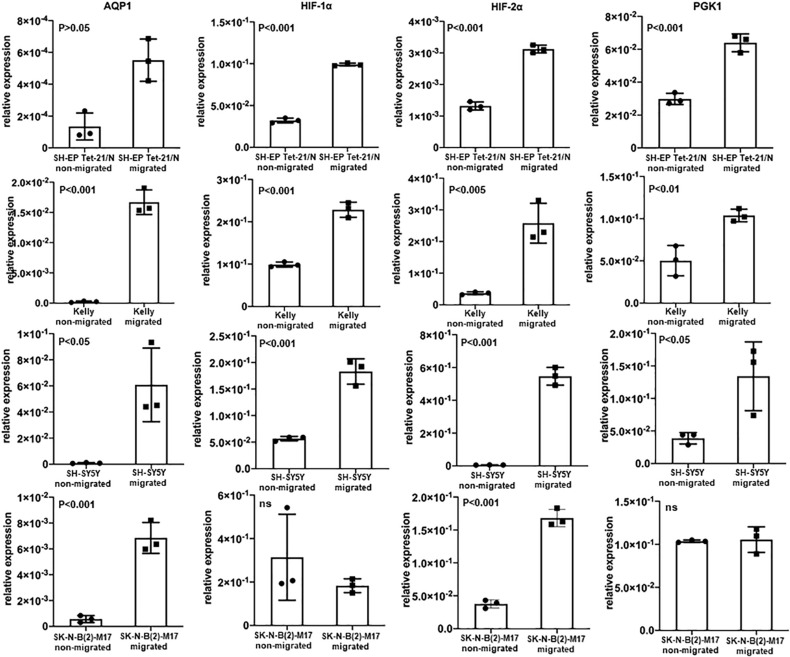
AQP1 expression of non-migrating and migrating cells and expression of hypoxia-related profile. Evaluation of AQP1 mRNA after selection of cells that have actually migrated and those which have not migrated in a transwell assay show an elevated expression of AQP1 in the migrated cells (first vertical column). This becomes very clear when looking at Kelly and SH-SY5Y cells. But also in cell lines SK-N-B(2)-M17 and SH-EP Tet-21/N, in which we did not detect large amounts AQP1 mRNA under hypoxia, there are significant differences between migrated and non-migrated cells, however with a lower relative expression (y-axis). A significantly increased expression can is also observed for HIF-1α (second vertical column), HIF-2α (third vertical column) and PGK1 (fourth vertical column) in migrated cell lines, with the exception of HIF-1α and PGK1 in SK-N-B(2)-M17. The experiment was performed twice, RNA triplicates were used for qPCR analysis each time and student’s *t*-test for analysis of data.

We modified the transwell assay to analyze properties of the migrating cells in the lower well compared to the non-migrating cells in the upper well. Messenger RNA was isolated from the harvested cells of upper and lower wells and quantitative PCR was performed. This way we could investigate properties of the migrating subset of cells. Through all cell lines the migrating cells showed high AQP1 expression as well as a hypoxia-related expression profile (Figure 3). AQP1 expression was especially low in cell lines SK-N-B(2)-M17 and SH-EP Tet-21/N, as was to be expected but nevertheless showed significantly increased AQP1 expression in the migrated cells. Migrated Kelly and SH-SY5Y however showed allover higher expression levels compared especially with SH-EP Tet-21/N cells. Cell line heterogeneity might result from different origin of these cell lines. This may result from great intra- and inter-tumor heterogeneity, differences in differentiation state and malignancy potential.

When looking at other hypoxia-related factors, specifically HIF-1α, HIF-2α, and PGK1, we observed an increased expression of these factors especially in Kelly and SH-SY5Y cells. Although the increase of HIF-2α is consistent in all cell lines, expression of HIF-1α and PGK1 is compromised in SK-N-B(2)-M17 and that of PGK1 in SH-EP Tet-21/N cells. As these experiments were performed under normoxic conditions, activation of HIF-1α, HIF-2α and PGK1 might only occur in the subgroup of migrating cells triggered by a transient hypoxic induction or due to other causes.

**FIGURE 4:**
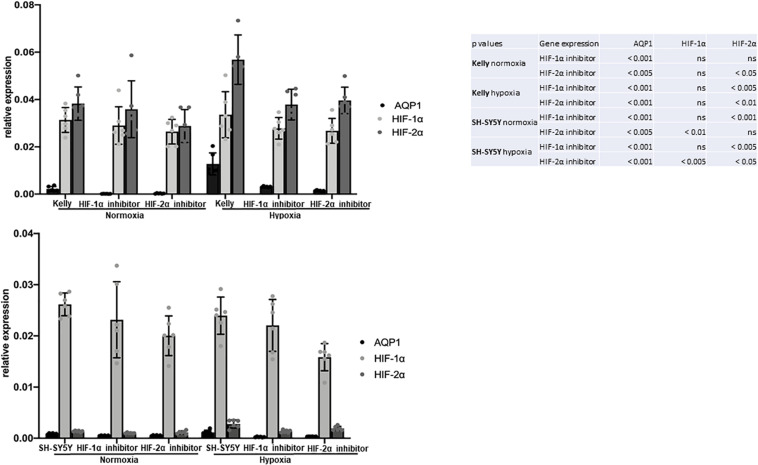
Attributes of migrating SH-SY5Y cells: Role of hypoxia. HIF-1α and HIF-2α inhibition of Kelly and SH-SY5Y cells in normoxia and after 6 h hypoxia. Relative expression of AQP1 mRNA shows that inhibition of HIF-1α and HIF-2α independently led to a decrease in AQP1 expression, more pronouncedly under hypoxic conditions in Kelly (upper panel) and in SH-SY5Y cells (lower panel). The experiment was performed twice with 3 RNA replicates each. One-way Anova was performed followed by Dunnett’s multiple comparison test using GraphPad Prism7. Normal distribution was confirmed. The error bars represent one standard deviation.

**FIGURE 5:**
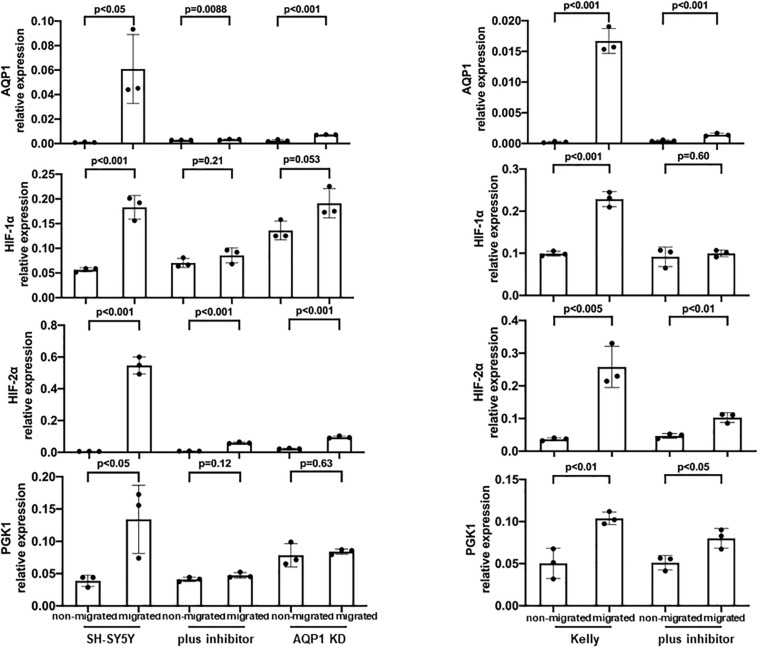
Inhibition of AQP1 and its effect on hypoxia-related factors. AQP1 mRNA expression of migrating SH-SY5Y and Kelly neuroblastoma cells is decreased through AQP1 inhibition by both the AQP1 inhibitor and in case of SH-SY5Y cells through the AQP1 knock-down construct. HIF-1α expression is not compromised in the knock-down and only partially under AQP1 inhibition. HIF-2α expression is down-regulated in the SH-SY5Y AQP1 knock-down and through AQP1 inhibition, while changes of PGK1 expression in either are not relevant. The experiment was performed twice, RNA triplicates were used for qPCR analysis each time and student’s *t*-test for analysis of data.

In Kelly cells inhibition of HIF-1α led to an only partial decrease of both HIF-1α and HIF-2α, while the inhibition of HIF-2α significantly decreased HIF-2α expression. Both, inhibition of HIF-1α and HIF-2α, led to a significantly decreased AQP1 mRNA expression in normoxia and in even more remarkably after hypoxia-induced HIF-response (Figure 4, upper panel). The same effect could be observed in SH-SY5Y cells. Moreover, in Kelly and SH-SY5Y cells inhibition of HIF-2α led to a significant decrease of HIF-2α expression, while reduction of HIF-1α expression through a HIF-1α inhibitor was apparent but not statistically significant (Figure 4, lower panel). A reason for this could be that the HIF-2α inhibition reacts on the gene expression level, while the HIF-1α inhibitor suppresses cellular HIF-1α without effecting transcription (Zimmer et al., 2008; Narita et al., 2009). Inhibition of the AQP1 protein in SH-SY5Y cells with TEA led to a decrease of migration as well as a reduction of AQP1 positivity on the mRNA level (Figure 5). The same effect was found in the SH-SY5Y AQP1 knock-down cell line compared to regular SH-SY5Y cells. While AQP1 is decreased in the knock-down cell line, HIF-1α, HIF-2α, and PGK1 are not or only partially decreased, as would be expected. We find however, that especially HIF-2α seems to be down-regulated by knock-down and inhibition of AQP1. There are no differences in PGK1 expression between migrated and non-migrated cells after knockdown or inhibition of AQP1. Multiple processes determine the regulation of HIF-1α which in itself is not very stable. Our data suggest that AQP1 is dependent on HIF-1α and HIF-2α, as has been hypothesized previously. It links these basic hypoxia-related factors to migratory processes in neuroblastoma.

## Discussion

AQP1 expression in neuroblastoma cells is up-regulated by hypoxic conditions. The increased AQP1 expression in our experiments enables the cells to form a more agile phenotype with migratory properties. This suggests that the hypoxic tumor microenvironment is the trigger for some tumor cells to transition into a migratory phenotype.

### Upregulation of AQP1 by Hypoxia

We show that AQP1 expression is up-regulated by hypoxia in neuroblastoma. This increase of AQP1 is preceded by an increase of HIF-1α protein, indicating that AQP1 expression could be regulated by HIF-1α. Abreu-Rodriguez and colleagues have shown that HIF-1α is involved in the promoter activation of AQP1 by hypoxia. HIF-1α however does not seem to be the only relevant transcription factor (Abreu-Rodriguez et al., 2011). A further study by Tanaka et al. (2011) showed that during the transcriptional activation of AQP1 by hypoxia the HIF-1α binding site of the 5′-promotor plays a key role in human retinal vascular endothelial cell (HRVEC). HIF-1α induced regulation of AQP1 has also been linked to edema in peripheral nerve injury in Schwann cells and after myocardial infarction (Zhang et al., 2013; Li et al., 2015). AQP1 inhibition has been proposed as a possible target for treatment of edema (Zhang et al., 2013). HIF-1α and several other hypoxia related genes such as pyruvate dehydrogenase 1 (PDK1), PGK1, carbonic anhydrase IX (CAIX) and prolyl hydroxylase 3 (PHD3) have been linked to high risk neuroblastoma (Qing et al., 2010; Dungwa et al., 2012; Ameis et al., 2013, 2016a, b). AQP1 and HIF-1α have also been associated with invasiveness and migration in neuroblastoma and other cancers as well as with resistance to chemotherapy (Hussein et al., 2006; Smith et al., 2010; Chen et al., 2015; Sun et al., 2017; Guan et al., 2018; Sato et al., 2018).

Together with an increased AQP1 expression in migrated tumor cells, we observe an increase of HIF-1α and HIF-2α in the migrated neuroblastoma cells. A different role in regulation of neuroblastoma has been suggested for HIF-2α (EPAS1- endothelial PAS domain-containing protein 1) (Noguera et al., 2009). While HIF-2α was identified to mark a subpopulation of immature neural crest-like perivascularly located cells associated with aggressive disease and distant metastasis in neuroblastoma (Pahlman and Mohlin, 2018), its role is still under discussion (Holmquist-Mengelbier et al., 2006). In this context it could be important to further investigate the role of AQP1 expression in neuroblastoma cancer stem cells, which might serve as an anti-tumor target (Mouhieddine et al., 2015; Bahmad et al., 2019, 2020). The interaction of HIF-2α with AQP1 seems to be multifaceted. A study by Ge et al. (2016) showed that AQP1 is necessary to stabilize HIF-2α in reperfusion after lung ischemia. Cellular co-expression of aquaporins 1,3, and 5 slows down natural HIF-2α degradation during prolonged hypoxia. AQP over-expression in PC12 cells prolonged HIF-2α stability during chronic hypoxia, leading to higher level of induction of its target genes and likely conferring to these cells a more tumor-like phenotype (Galan-Cobo et al., 2013).

### Increased Water Permeability and Migration in AQP1-Expressing Neuroblastoma Cells

Our experiments show that AQP1 changes in the modified tumor cells are functional and lead to an enhanced or decreased water transport of neuroblastoma cells. This indicates that AQP1 can serve as facilitator of migration. AQP1 has been linked to several physiological and pathological processes reaching from water homeostasis, e.g., in the intestine, in follicular cells and many others to cell proliferation and migration (Pini et al., 2017; Skowronski et al., 2017; Zhu et al., 2017; Hua et al., 2019). A model of AQP1 dependent cell migration reported by Verkman et al. is based on the increased water influx into the tumor cell that facilitates restructuring of intracellular actin filaments and the reshaping of tumor cells to pass through confined spaces (Saadoun et al., 2005; Papadopoulos et al., 2008). A “water engine” model by Stroka et al. (2014) suggests an increased mobility of cells through increased water in- and efflux.

The increased AQP1 expression in our experiments enables the cells to form a more agile phenotype with migratory properties. It has been shown that AQP1 furthers lung metastases in a breast cancer model and lead to an increased metastatic potential in melanoma and glioma (Saadoun et al., 2002; Papadopoulos et al., 2008; Esteva-Font et al., 2014). AQP1 deletion can inhibit migration of tumor cells (Saadoun et al., 2005). Therefore, AQP1 has been suggested as a possible therapeutic target for several tumors including tumors of the central nervous system as well as other solid tumors like melanoma or colon cancer (Deb et al., 2012; Wei and Dong, 2015; Dorward et al., 2016; Jagirdar et al., 2016; Pei et al., 2016; Wang et al., 2017; Simone et al., 2018; Yang et al., 2018). Several inhibitors have been shown to effectively suppress AQP1 function and lead to reduced cell migration and induction of apoptosis (AqB013 and AqB050) reduced cell viability, increase of apoptosis, reduced migration and tube formation (Bacopaside II), reduced migration without affecting viability (AqB011) (Kourghi et al., 2016; Palethorpe et al., 2018; Tomita et al., 2019). The bumetanide derivatives AqB007 and AqB011 represent selective blockers of AQP1 ion channel conductance (Kourghi et al., 2016). Further chemical compounds have been tested as AQP1 inhibitors (Mola et al., 2009). Amongst the quaternary ammonium-based compounds TEA (tetraethylammonium) is the lead component in blocking AQP1. It is more selective for AQP1 than for potassium channels and is highly effective regarding water permeation (Detmers et al., 2006). TEA potently and reversibly inhibits water permeation without influencing AQP1 expression levels (Detmers et al., 2006; Brooks et al., 2000).

### Attributes of Migrating Tumor Cells

Neuroblastoma cells with a high AQP1 expression show a distinct enhanced migratory pattern compared to cells with low AQP1 expression. In the moving cells AQP1 is prominently expressed in the lamellipodia as shown in Figures 2D–F. Actin is locating to these outreaching cell parts and facilitates reshaping of the forward moving cell. Saadoun et al. (2005) could describe this polarization of AQP1 protein to the lamellipodia in migrating CHO cells and first suggested the model an enhanced water influx through AQP1 expression. The enhanced water influx facilitates restructuring of the actin filaments within the cell cytoskeleton, reshaping the cell and enhancing migration though a forward pull (Saadoun et al., 2005).

In our migration experiments these migrating tumor cells exhibit a different mRNA expression phenotype compared to non-migrating tumor cells. Throughout the tested cell lines AQP1 is highly expressed in migrating but not in non-migrating cells. This effect can be suppressed by inhibiting AQP1 either with an inhibitor or through a knock-down cell construct. The elevated AQP1 expression in the migrating cells is combined with a more hypoxic expression profile with elevated HIF-1α and HIF-2α levels. In a study by Westerlund et al. (2017) high levels of HIF-1α but not of HIF-2α correlate with neural differentiation genes and better prognosis, but negatively correlate with key high risk features (e.g., NMYC). This suggested an unanticipated tumor-suppressive role for HIF-2α in neuroblastoma. Another study demonstrates that although HIF-1α is preferentially expressed in NMYC amplified neuroblastoma cells there is no defined regulatory correlation between these two factors (Qing et al., 2010). Further studies in neuroblastoma showed that HIF-2α but not HIF-1α was strongly expressed in well vascularized areas (Holmquist-Mengelbier et al., 2006). While reduced oxygen levels (5% O_2_) led to HIF-2α stabilization transcribing to vascular endothelial growth factor (VEGF), only further reduced oxygen levels (1% O_2_) led to HIF-1α stabilization with an acute response while HIF-2α accumulated and governed hypoxic gene activation (Holmquist-Mengelbier et al., 2006). HIF-2α seems to be restricted to neural cell-derived tumors. Hypoxia appeared to lead to *de novo* transcription of HIF-2α mRNA rather than an increased mRNA stability (Hamidian et al., 2015). Our data suggest that expression of AQP1 is linked to HIF-1α as well as HIF-2α and that this hypoxia-related profile is linked to migratory processes in neuroblastoma.

## Conclusion

Migrating tumor cells express elevated AQP1 levels and a hypoxic biochemical phenotype. Expression of AQP1 can functionally facilitate tumor cell migration and is up-regulated by hypoxia. Elevated AQP1 might be a key driver in transitioning stable tumor cells to migrating tumor cells in a hypoxic microenvironment.

With regard to a clinical perspective, these findings provide the foundation for the inhibition of metastatic spread by targeting of AQP1-facilitated migration and of hypoxia-induced transitioning of cells to a more aggressive phenotype.

## Data Availability Statement

The raw data supporting the conclusions of this article will be made available by the authors, without undue reservation, to any qualified researcher.

## Author Contributions

ZH and SG: conceptualization. ZH, ML, UK, and SG: methodology. ZH, ML, UK, CP, and SG: validation. ZH, ML, and SG: formal analysis and writing—original draft preparation. SG, CP, and SH-C: resources. ZH, ML, SG, CP, and SH-C: writing—review and editing. SG: supervision and project administration. SG, ZH, CP, and ML: funding acquisition. All authors have read and agreed to the published version of the manuscript.

## Conflict of Interest

The authors declare that the research was conducted in the absence of any commercial or financial relationships that could be construed as a potential conflict of interest.

## Footnotes

1 https://hgserver1.amc.nl/cgi-bin/r2/main.cgi

## Supplementary Material

Supplementary Video 1 Water permeability induced cells shape changes of SH-SY5Y cells. The video shows the reaction of SH-SY5Y cells to an osmotic stimulus in confocal microscopy (left lower panel: fluorescence at 488 nm; upper right panel: phase contrast; lower right panel: overlap). The film demonstrates the enormous shape changes enhancing their ability to move through confined spaces. This requires major water fluxes across the membrane, which are greatly facilitated by the presence of aquaporin. Although the water efflux is not solely due to aquaporin 1 water channels, we show in the stopped-flow spectroscopy (Figure 2B) that aquaporin expression is functional and accelerates water flux.
