# Supplementary material for: AQP1 Is Up-Regulated by Hypoxia and Leads to Increased Cell Water Permeability, Motility, and Migration in Neuroblastoma
Source: Front Cell Dev Biol. 2021 Feb 11;9:605272. doi: 10.3389/fcell.2021.605272 (PMC7905035; doi:10.3389/fcell.2021.605272)

Supplementary Data: Expression of NMYC

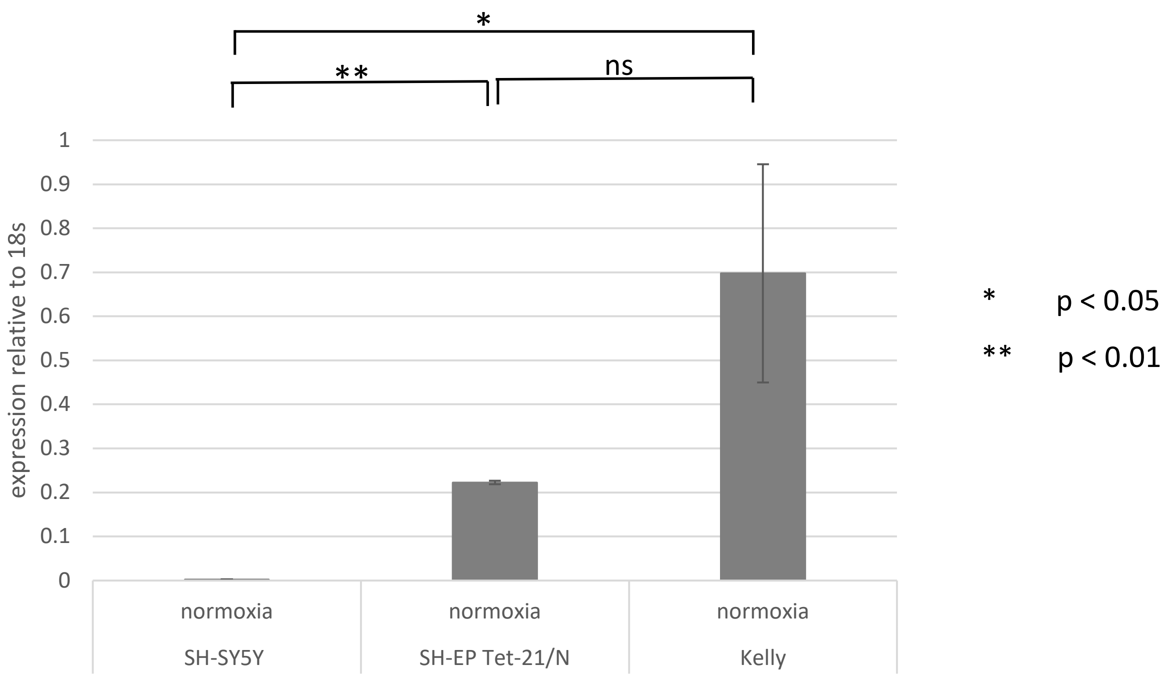

Supplementary Data: Expression of AQP. Comparison between cell lines after migration

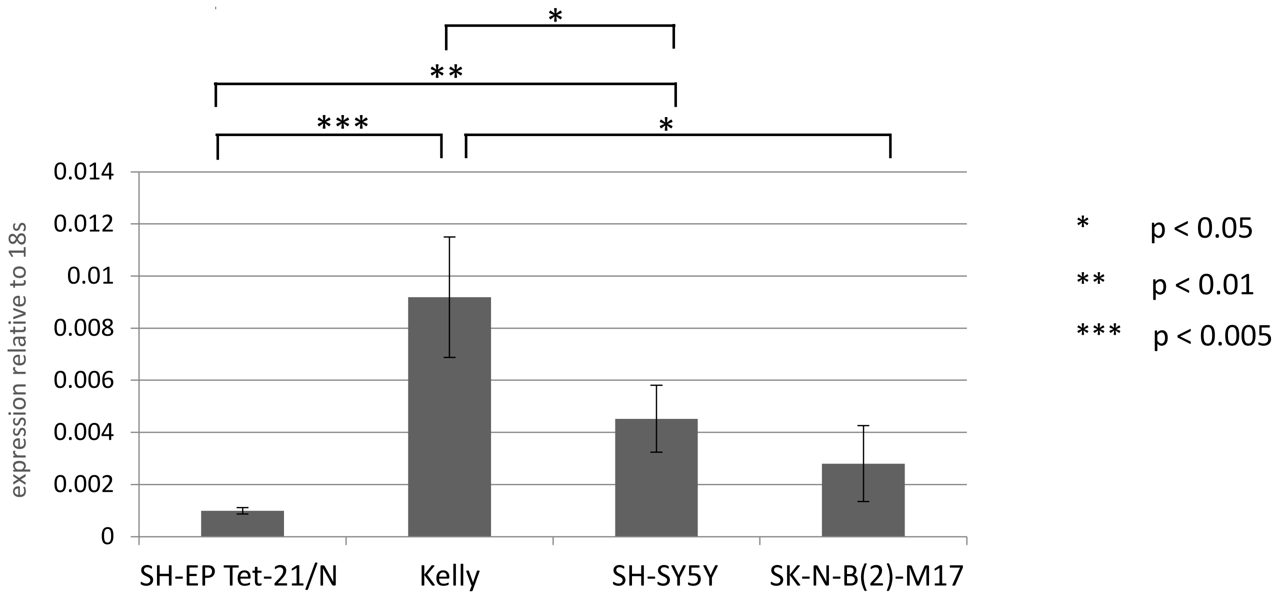

Figure 1C Western Blot Data

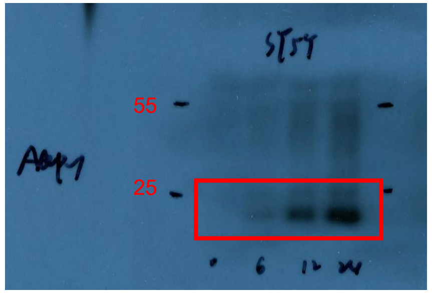

SH-SY5Y AQP1 expression

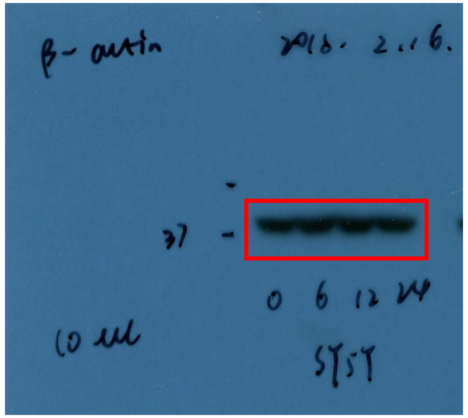

SH-SY5Y β-actin expression

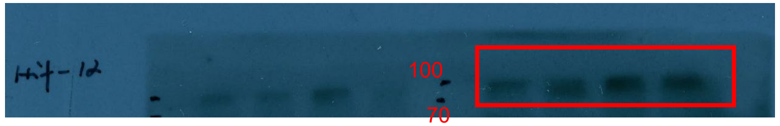

SH-SY5Y Hif-1α expression

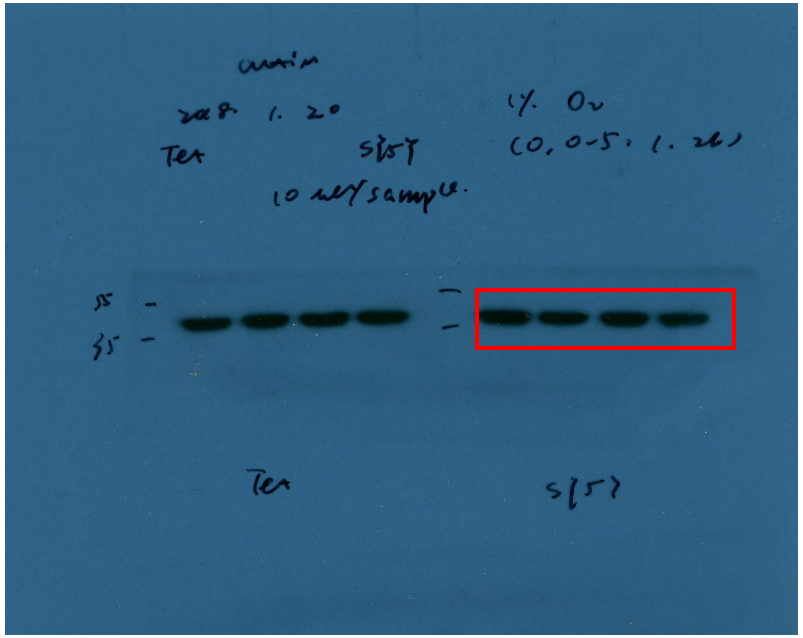

SH-SY5Y β-actin expression

Figure 1C Western Blot Data

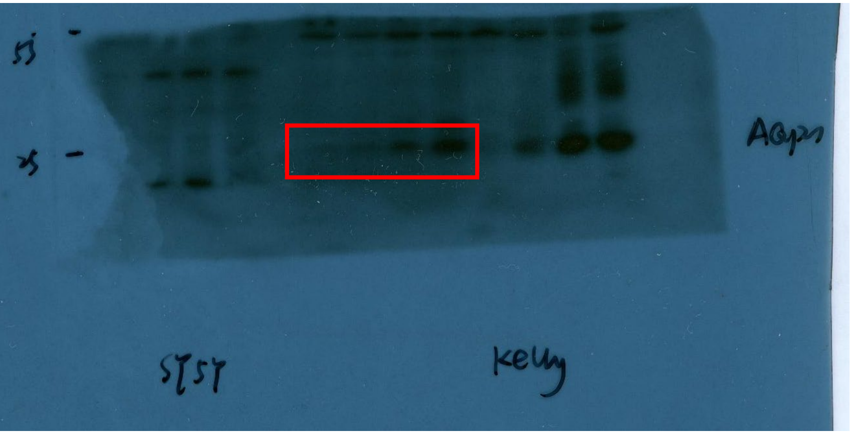

Kelly AQP1 expression

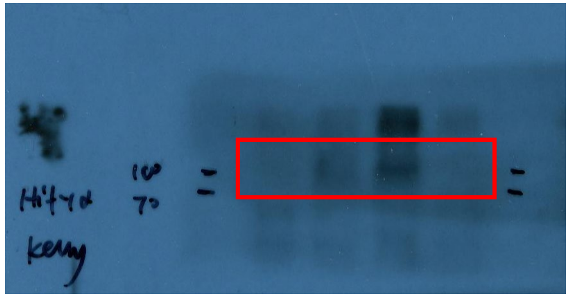

Kelly Hif-1α expression

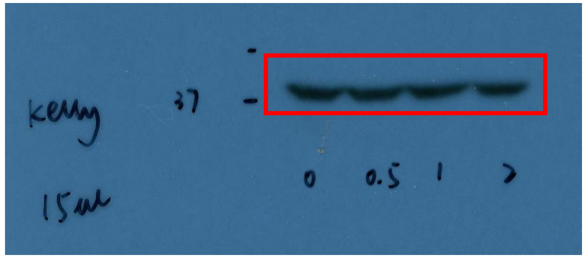

Kelly β-actin expression

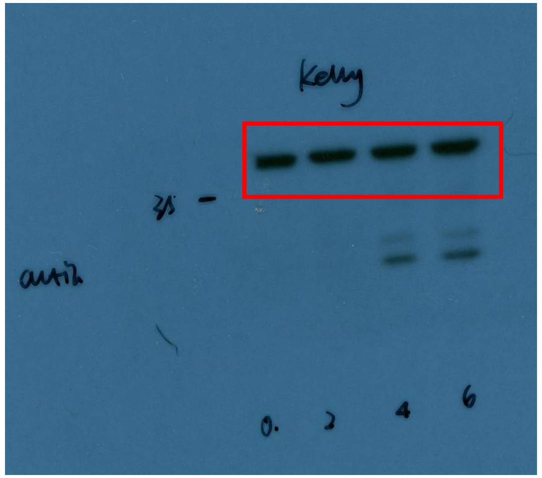

Kelly β-actin expression

Grey scan

AQP1 and HIF-1α relative to β-actin (analysis performed using ImageJ software and plotted using GraphPad Prism software

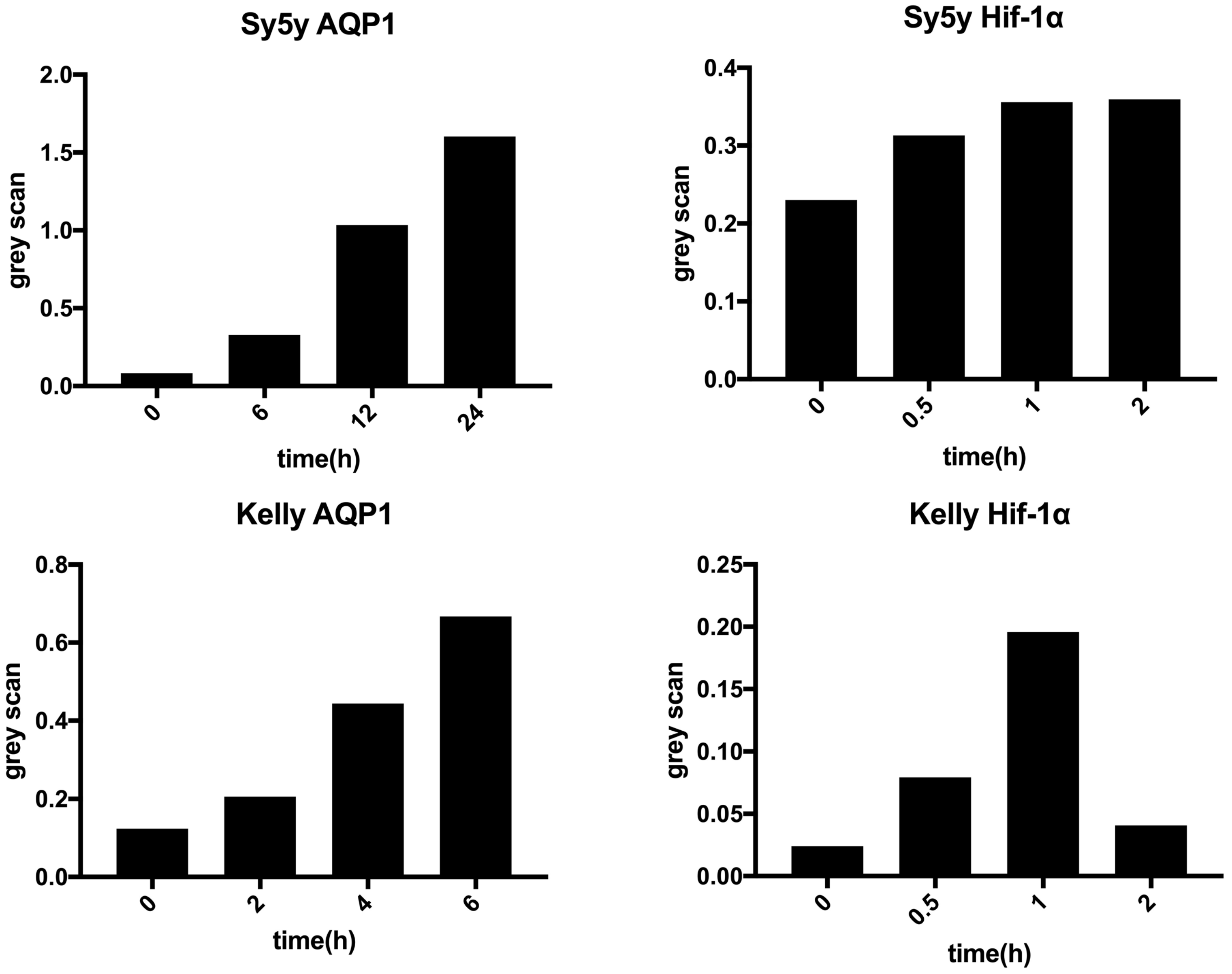

Figure 2A Western Blot Data

Loading scheme:
x_x_x_x_M_SH-SY5Y (normoxia)_SH-SY5Y(hypoxia)_SH-SY5Y AQP1knockdown_SH-SY5Y AQP1overexpression_M

AQP1 30sec, 1min and 3 min exposure

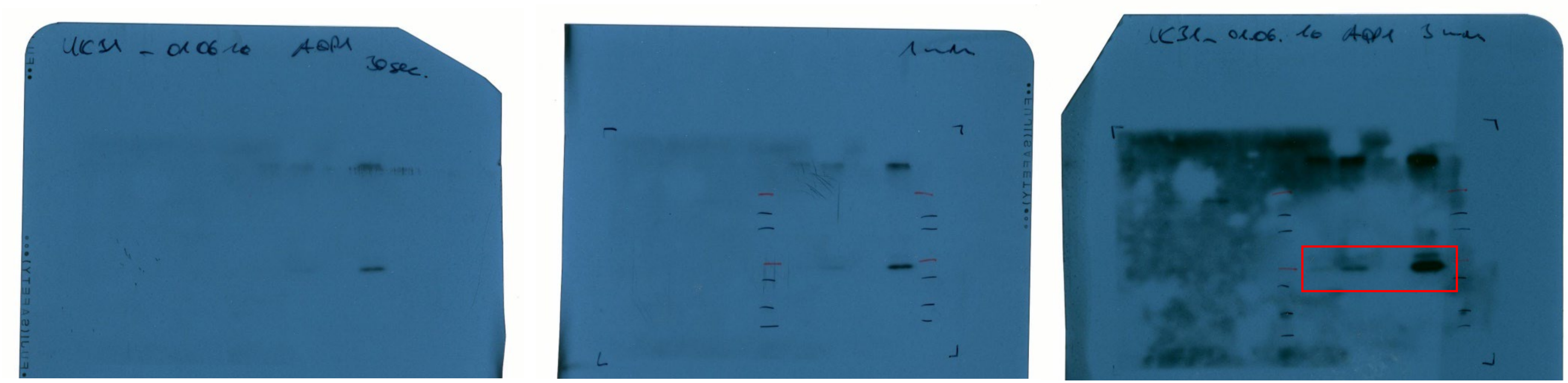

βactin 30sec, 1min and 3 min exposure

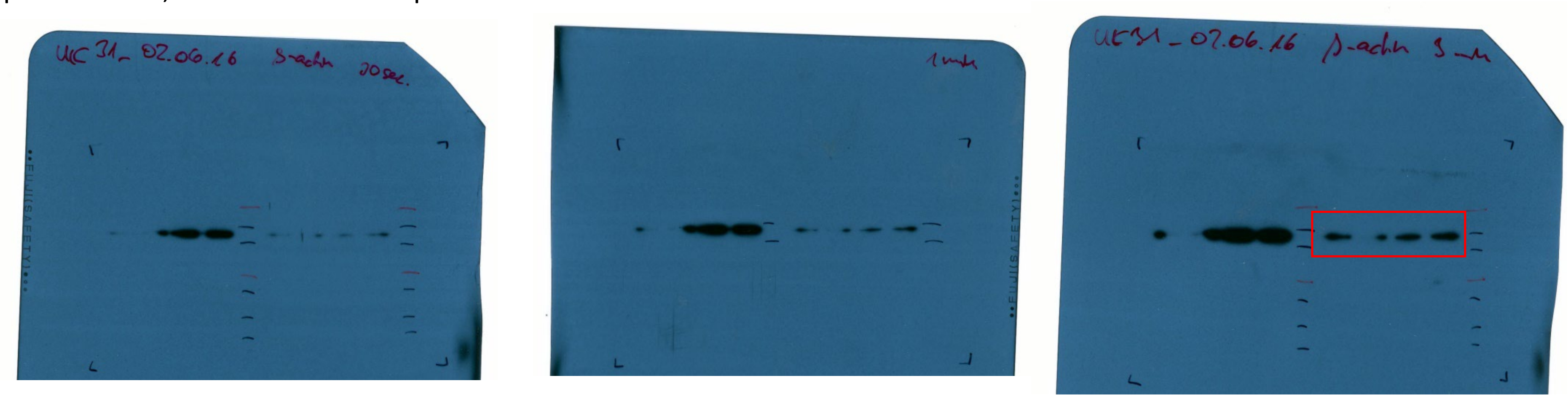

Grey scan

AQP1 relative to β-actin (analysis performed using ImageJ software and plotted using GraphPad Prism software

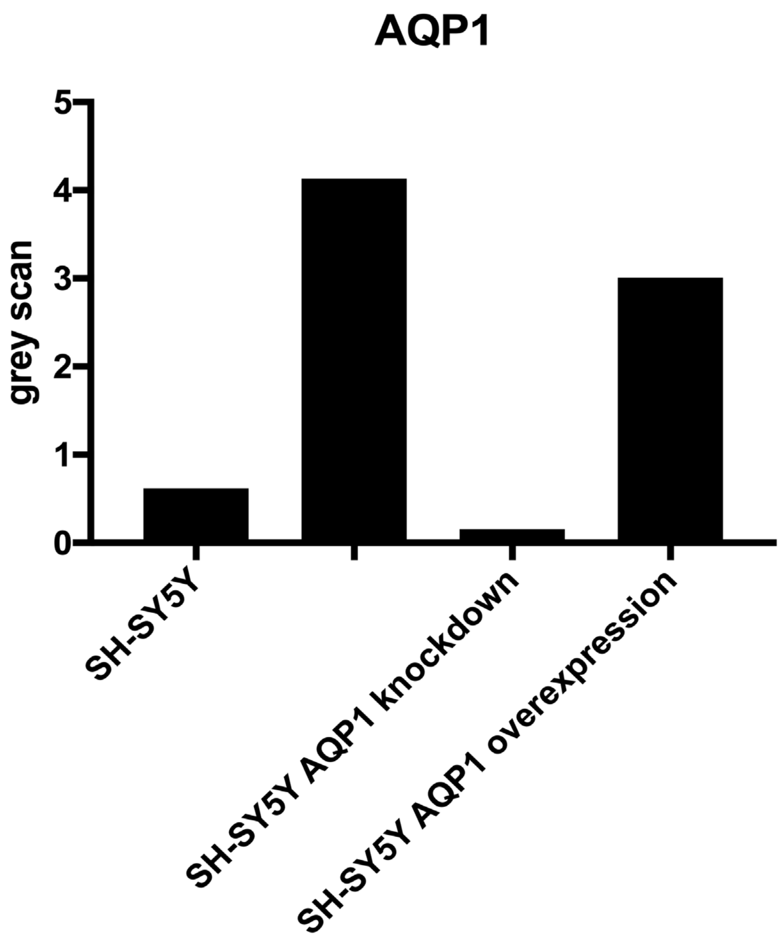

Supplement: Supplementary file 2 [file Data_Sheet_1.PDF]
